# Ichnologic and sedimentologic datasets from the Ediacaran–Cambrian Chapel island formation, Newfoundland, Canada

**DOI:** 10.1016/j.dib.2024.111258

**Published:** 2024-12-24

**Authors:** Romain Gougeon, M. Gabriela Mángano, Luis A. Buatois, Guy M. Narbonne, Brittany A. Laing, Maximiliano Paz

**Affiliations:** a Department of Geological Sciences, University of Saskatchewan, Saskatoon, SK S7N 5E2, Canada; b Geo-Ocean, University of Brest, CNRS, Ifremer, UMR 6538, Plouzané F-29280, France; c Department of Geological Sciences and Geological Engineering, Queen's University, Kingston, ON K7L 3N6, Canada

**Keywords:** Trace fossils, Bioturbation, Sedimentary facies, Cambrian explosion

## Abstract

Extensive ichnologic and sedimentologic datasets were gathered from six localities (Fortune Head, Fortune North, Grand Bank Head, Lewin's Cove, Little Dantzic Cove, and Point May) of the Ediacaran–Cambrian Chapel Island Formation at Burin Peninsula, southeastern Newfoundland, eastern Canada. 1708.2 m of sedimentary strata were logged at a centimeter scale (1:40) using a Jacob staff, in addition to 11.08 m of strata reported at a millimeter scale (1:1.67). Sedimentary logs focus on: (1) bed geometry; (2) bed thickness; (3) bed grain size; (4) sandstone/ mudstone ratio; and (5) sedimentary structures. For each log, trace-fossil datasets were reported, consisting of: (1) bioturbation intensity in cross-section (1596 data points); (2) bed surface bioturbation intensity (1481 data points); (3) stratigraphic position of individual trace fossils; (4) trace-fossil width (3164 data points); (5) trace-fossil depth (1539 data points); and (6) ichnotaxonomic classification (3510 trace fossils identified at ichnospecies rank). The datasets are of importance to researchers interested in the palaeoecological signals depicted in this classic Ediacaran–Cambrian succession or in the compilation of worldwide data for deciphering macro-evolutionary trends in early animal life.

Specifications Table

**Table:** 

Subject	Earth and Planetary Sciences (Geology, Palaeontology, Stratigraphy)
Specific subject area	The early evolution of animal life based on trace-fossil evidence preserved in the stratigraphic record of the Chapel Island Formation (CIF) [[1], [2], [3], [4], [5]].
Type of data	Chart, Figure Raw
Data collection	The dataset was first reported on field books and later digitalized on Adobe Illustrator. Sedimentologic data were gathered using a Jacob staff from naked-eye observations in the field. Sixty-four samples were collected, slabbed and polished. Bioturbation intensity in cross-section was evaluated in the field using a Bioturbation Index (BI) [6] and bed surface bioturbation intensity using a Bedding Plane Bioturbation Index (BPBI) [7]. Trace-fossil metrics were reported in the field using a manual caliper. Trace-fossil taxonomy was first evaluated in the field, and later supported by a monographic work [1]. About 20,000 photographs were taken to refine any sedimentologic or ichnologic observation.
Data source location	Outcrops are located in sea cliffs of Burin Peninsula, southeastern Newfoundland, eastern Canada (Fig. 1). Closest towns to the outcrops are Fortune, Grand Bank, Lewin's Cove, and Point May. Latitude and longitude for collected samples/data are: + Fortune Head: 47°04′32″N 55°51′19″W + Fortune North: 47°04′42″N 55°49′56″W + Grand Bank Head: 47°06′26″N 55°46′03″W + Lewin's Cove: 47°04′30″N 55°12′18″W + Little Dantzic Cove: 46°57′33″N 55°59′13″W + Point May: 46°54′19″N 55°56′57″W
Data accessibility	Repository name: Mendeley Data Data identification number: 10.17632/g2g74cj4x7.1 Direct URL to data: https://data.mendeley.com/datasets/g2g74cj4 × 7/1
Related research article	R. Gougeon, M.G. Mángano, L.A. Buatois, G.M. Narbonne, B.A. Laing, M. Paz, Ichnology of the Ediacaran-Cambrian Chapel Island Formation of Newfoundland, Canada: unraveling bioturbation at the onset of the Cambrian Explosion, Fossils and Strata. In press.

## 1 Value of the Data

• The data represent the first modern comprehensive compilation of information from the CIF. Previous sedimentologic investigations were done during the 1980′s (e.g. Myrow [8]). Ichnologic work started in the 1980′s as well (e.g. Narbonne et al. [9]), and subsequent studies focused on very specific aspects (e.g. the preservation-style of burrows in Droser et al. [10], or the palaeoecologic signal of early Fortunian trace fossils in Laing et al. [3]).
• The data are also useful because the CIF hosts the type section of the Cambrian at Fortune Head based on an ichnostratigraphic scheme [1,9]. This decision led to some debate [11] and this database is therefore necessary to assess this scheme.
• The data will be beneficial to any researcher interested in early animal evolution. The dataset provides the most up-to-date reports on trace-fossil metrics that are necessary to untangle macro-evolutionary trends at the onset of the Cambrian explosion. The data also supply extensive sedimentary facies characterization that is essential to decipher environmental controls and sea-level changes.
• The data will be beneficial to any researcher interested in revisiting the succession in the future, or for the preparation of conference field excursions that are common in this area (e.g. field parties visited the succession during the 3rd Ichnia conference in 2012, and for the International Symposium on the Ediacaran-Cambrian Transition in 2017).
• The data are used as a basis for revising the ichnotaxonomy and depositional environment interpretation of the succession (Gougeon et al. [1]). They were also used to design time-environment matrices to discriminate temporal from evolutionary controls on trace fossils in the succession, which led to the identification of global palaeoecological stages (Gougeon et al. [5]).
• The data can serve as a template for standardized sedimentologic and ichnologic data collection to researchers interested in revisiting similar Ediacaran–Cambrian successions worldwide, and to anybody studying other successions from different time periods or depositional settings.

## 2 Background

The CIF is renowned worldwide for its trace-fossil content and continuous exposure of siliciclastic strata, but the decision to place the Ediacaran–Cambrian boundary at the first appearance of the trace fossil *Treptichus pedum*, also delineating the base of the *T. pedum* Ichno-Assemblage Zone [9,12], led to debates in the research community. Sedimentologic and ichnologic work in the succession started in the 1980′s, and subsequent trace-fossil studies were dedicated to specific aspects and to restricted stratigraphic intervals of the succession. The objectives of this dataset are to: (1) provide the most extensive sedimentologic and ichnologic data from the CIF to date; (2) supply the necessary background information to characterize sedimentary facies and revise ichnotaxonomy (Gougeon et al. [1]); (3) allow for macro-evolutionary analyses by discriminating environmental versus evolutionary controls on trace-fossil distribution (Gougeon et al. [1,4,5]); and (4) serve as supportive information for any future investigation of the CIF.

## 3 Data Description

**Fig. 1:**
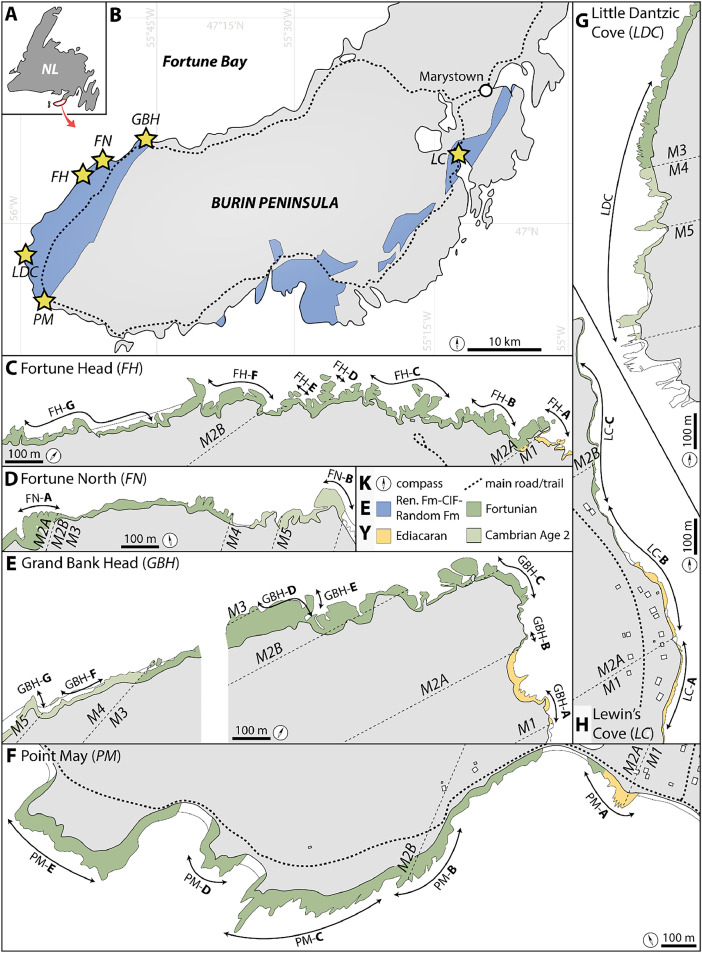
Location maps of the six studied outcrops. The figure is reproduced from figure 4 in Gougeon et al. [1]. Abbreviations: NL, Newfoundland; M1 to M5, Members 1 to 5 of the Chapel Island Formation; Ren. Fm, Rencontre Formation; CIF, Chapel Island Formation; and Random Fm, Random Formation. **A.** General map of Newfoundland, eastern Canada. **B.** General map of Burin Peninsula showing location of outcrops (stars) exposed along the coast. **C–G.** Close-ups at each outcrop showing exposed areas and measured sedimentologic logs.

There are two data files in the repository attached to this article:

+ The first file entitled “Data 1” is 102 pages long and corresponds to centimeter-scale logs reported at 1:40. These cover 1708.2 m of strata and were measured at Fortune Head, Fortune North, Grand Bank Head, Lewin's Cove, Little Dantzic Cove, and Point May (Fig. 1). A front page provides page numbers for the different elements of the dataset. Pages 2 and 3 correspond to a detailed legend. The file then continues with seven sections from Fortune Head (FH-A to FH-H, from pages 4 to 29), two sections from Fortune North (FN-A and FN-B, from pages 30 to 36), six sections from Grand Bank Head (GBH-A to GBH-G, from pages 37 to 60), three sections from Lewin's Cove (LC-A to LC-C, from pages 61 to 68), one section from Little Dantzic Cove (LDC, from pages 69 to 85), and five sections from Point May (PM-A to PM-E, from pages 86 to 102).

+ The second file entitled “Data 2” is 21 pages long and is composed of millimeter-scale logs reported at 1:1.67. These represent 11.08 m of strata and were recovered from Fortune Head (four sections 1.40 m, 1.40 m, 1.00 m, and 1.40 m thick), Grand Bank Head (three sections 0.94 m, 0.80 m, and 1.71 m thick), Little Dantzic Cove (two sections 0.93 m and 1.00 m thick), and Point May (one section 0.50 m thick). Page 1 is a front page providing page numbers for the different elements of the dataset. Page 2 consists of an additional legend (other legends can be found on pages 2 and 3 of the “Data 1” file). Pages 3 to 21 correspond to the 10 stratigraphic sections recovered.

## 4 Experimental Design, Materials and Methods

### 4.1 Materials: outcrops and stratigraphic characteristics

**Fig. 2:**
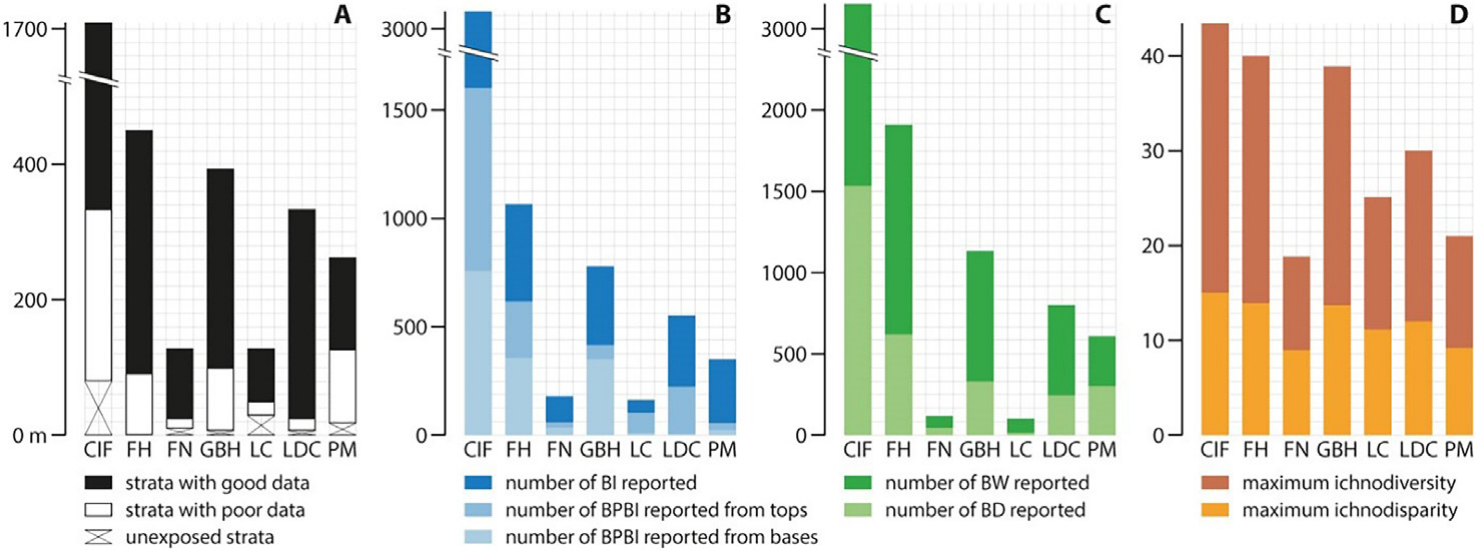
Comparison of datasets for the entire Chapel Island Formation (CIF) and the six individual localities (FH, Fortune Head; FN, Fortune North; GBH, Grand Bank Head; LC, Lewin's Cove; LDC, Little Dantzic Cove; PM, Point May). CIF data are either defined by the sum of data from the six localities (for **A, B**, and **C**), or are based on a composite succession (for **D**; see figure 3 in Gougeon et al. [1] for detail on the composite succession). **A.** Stratal extension. **B.** Numbers of Bioturbation Index (BI) and Bedding Plane Bioturbation Index (BPBI) data points reported. **C.** Numbers of Burrow Width (BW) and Burrow Depth (BD) data points reported. **D.** Maximum ichnodiversity and ichnodisparity.

The six outcrops are exposed in coastal cliffs at Burin Peninsula, southeastern Newfoundland, eastern Canada (Fig. 1). Outcrops were selected based on the extension of their stratigraphic record, the quality of their sedimentologic and ichnologic data, and their convenient access. These localities provide stratigraphic records that cover the full extension of the CIF (ca*.* 1000 m thick) and its five informal members (M1 to M5) [1,8,9]. Fig. 1C–G details the stratigraphic extension of each section reported in the “Data 1” file. Summary and comparison of the stratigraphic and ichnologic datasets from the six localities are available in Table 1 and Fig. 2.

**Table 1:** Summary of datasets for each locality. Chapel Island Formation (CIF) data (far right column) are either defined by the sum of data from the six localities (for stratigraphic thicknesses, bioturbation indices, and burrow widths and depths), or are based on a composite succession (for maximum ichnodiversity and ichnodisparity; see figure 3 in Gougeon et al. [1] for detail on the composite succession).

	Fortune Head	Fortune North	Grand Bank Head	Lewin's Cove	Little Dantzic Cove	Point May	CIF
Strata with good data	360.8 m	99.3 m	294.9 m	74.1 m	309.9 m	134.1 m	1273.1 m
Strata with poor data	90.3 m	14.4 m	91.8 m	21.5 m	6.8 m	107.4 m	332.2 m
Unexposed strata	0 m	9.3 m	6.8 m	28.3 m	16.9 m	18.6 m	79.9 m
Total stratigraphic thickness reported	451.1 m	123.0 m	393.5 m	123.9 m	333.6 m	260.1 m	1708.2 m
BI reported	446	123	357	53	324	293	1596
BPBI reported from bed tops	259	26	69	101	227	32	714
BPBI reported from bed bases	357	35	348	3	1	23	767
Total bioturbation indices reported	1062	184	774	157	552	348	3077
BW reported	1316	799	555	85	89	320	3164
BD reported	604	334	249	46	11	295	1539
Maximum ichnodiversity	26	10	25	14	18	12	28
Maximum ichnodisparity	14	9	14	11	12	9	15

Fortune Head (FH) is renowned for hosting the Cambrian type section since 1992 [12]. FH is located 1.8 km to the west of the town of Fortune and is easily accessible by car from Highway 220 and through a well-maintained trail. Strata at FH were observed from base (northeast) to top (southwest) in seven sections that do not display stratigraphic overlap. FH-A (i.e. section A at Fortune Head) is 34.1 m thick and covers upper M1 and M2A strata. FH-B is 93.3 m thick and covers M2A strata up to an interval dominated by red-colored bedding. FH-C is 153.7 m thick and covers the overlying M2A strata. FH-D corresponds to a small interval, 6.7 m thick, that was more difficult to access and that is not in direct stratal continuity with the underlying FH-C and overlying FH-E sections because of important faulting in the area [8]. FH-D records the transition from the top of M2A to the base of M2B. FH-E is 13.2 m thick and covers M2B strata. FH-F is 89.8 m thick and corresponds to M2B strata as well. Finally, FH-G is a section with poorer exposure, 63.2 m thick, consisting of the uppermost record of M2B strata at FH. These seven sections are composed of thinly laminated to thin-bedded, medium mudstone and very fine- to medium-grained sandstone. They are mostly silver-green, green, and grey in color, but with red beds at intervals.

Fortune North (FN) is exposed 400 m north of the town of Fortune. The top of the succession (M5, to the east) can be accessed easily thanks to a trail running into a nearby beach. However, the base of the succession (M2A, to the west) is more difficult to reach as there is no trail passing nearby, and access from the top of the cliff is complicated because of the cliff steepness. Landing et al. [13] managed to reach an interval covering M3 and M4 strata by boat, an area that was not investigated in our study. Two sections were recovered at FN. FN-A is 45.8 m thick and is composed mostly of M2A strata, with the uppermost 3.6 m belonging to M2B. This interval is made of heterolithic medium mudstone and thinly laminated to thin-bedded, very fine- to medium-grained sandstone. M2A strata is silver-green and grey in color, whereas M2B strata is red. FN-B is 76.9 m thick and corresponds to M5 strata. The succession is mostly composed of red and green thin- to very thick-bedded, very fine- to fine-grained sandstone with rare medium mudstone.

Grand Bank Head (GBH) is coeval with FH but with thicker M1 exposures and partial exposures of M4 and M5. GBH crops out around Grand Bank Cape, which is located 500 m northwest of the town of Grand Bank. M1 to M2B strata (to the southeast) are easily accessible by the Marine Hike trail which starts at Admiral Cove, northwest of Grand Bank, and runs all around the cape. M4 and M5 strata (to the northwest) are better accessed from the other end of the Marine Hike trail, which intersects Highway 220 2.5 km to the west of Grand Bank. Seven sections were recovered from GBH. GBH-A is 64.1 m thick and is composed of M1 strata that are in continuity with the underlying Rencontre Formation. GBH-B is a small interval, 13.7 m thick, that could only be reached by boat. It is made of M2A strata that is not in continuity with GBH-A and GBH-C [1,4]. GBH-C is 139.5 m thick, and covers M2A and M2B strata. GBH-D is 62.6 m thick, covering M2B strata but with multiple intervals lacking good exposures for data collection. GBH-E is a section 27.0 m thick and consists of M2B strata. This section is in geographic proximity to GBH-D (Fig. 1E), possibly separated by a fault, but their relationship – whether in stratal continuity or coeval – is unclear, and we follow Myrow [8] in keeping GBH-E as a separate unit. GBH-F is 46.3 m thick and represents M4 strata, with the uppermost 1.1 m corresponding to the base of M5. GBH-G is 40.3 m thick representing middle M5 strata. GBH-F and G are not in stratal continuity with previous sections as the intervening interval was either not accessible or not exposed at all. At GBH, M1 to M2B strata are typically heterolithic, red, silver-green, or grey, made of medium mudstone and thinly laminated to thin-bedded, very fine- to medium-grained sandstone. M4 strata is composed of fine mudstone, with in places limestone nodules and rare limestone beds. M5 strata is made of green thin- to thick-bedded, very fine- to fine-grained sandstone with rare medium mudstone.

Lewin's Cove (LC) is the only outcrop located on the east coast of Burin Peninsula, 1.1 km to the south of the town center. Highway 220 passes nearby the outcrop, but because vegetation is in places dense, access is limited to a few docking areas for boats and private trails. In addition, as the tidal range within the cove is more important than at any other locality, access is favored at low tides, but still the rock exposures are narrow because of continuous coastal erosion from waves and tides (Gougeon et al. [4]). Three sections were recovered at LC, from base (south) to top (north). LC-A is 29.4 m thick and corresponds to M1 strata. LC-B is 48.9 m thick, with some intervals covered by vegetation or poorly exposed, and consists of M1 to M2A strata. LC-C is 45.6 m thick and covers M2A strata. The three sections are made of heterolithic medium mudstone and thinly laminated to thin-bedded, very fine-grained sandstone. Deposits are mostly silver-green and grey, in places with red intervals in LC-A and LC-C as well.

Little Dantzic Cove (LDC) is located on the southwestern coast of Burin Peninsula, ca*.* 15 km southward of Fortune and ca*.* 5.5 km northward of Point May. It is the most difficult outcrop to access, as Highway 220 passes 2.7 km inland from the investigated strata without connecting road or trail. The most convenient access is by parking the car at Pieduck Point and hiking along the coastline northward for ca*.* 2 km. Strata at LDC is only composed of one section oriented north (base) to south (top), 334.1 m thick, that encompasses M3, M4, and M5 continuously. M3 strata is dominated by silver-green and grey medium mudstone with thinly to thickly laminated, very fine- to fine-grained sandstone. M4 strata is made of red, purple, grey, and green fine mudstone with rare thinly laminated, very fine- to fine-grained sandstone. M5 strata is composed in its lower interval by heterolithic grey-green sandy mudstone with thinly laminated to thin-bedded, very fine- to medium-grained sandstone, and in its upper interval by red and green, thin- to very thick-bedded, very fine- to fine-grained sandstone.

Point May (PM) is exposed close to a beach located northwest of the town center. Access from Lories hamlet is the most convenient. The base of the outcrop is oriented to the south. The top, towards north, can be reached by walking along the coastline on rocks or by using a walking trail atop the cliff. Five sections define PM strata. PM-A is 52.8 m thick and comprises M1 and M2A strata. PM-B is 24.2 m thick, covers M2A and M2B strata, and is separated from PM-A by a gap of rock exposure. PM-C (142.2 m thick), PM-D (35.4 m thick), and PM-E (25.5 m thick) all represent M2B strata. In all sections, covered intervals are common, but the exposed deposits are characterized by red, silver-green, and grey, medium mudstone and thinly laminated to thin-bedded, very fine- to medium-grained sandstone.

### 4.2 Materials: trace and body fossils

Trace fossils are found throughout the six outcrops, although with variations in diversity and abundance (see Gougeon et al. [1] for details). Trace fossils correspond to twenty-eight ichnogenera and fifty-two ichnospecies, which are cf. *Allocotichnus dyeri, Archaeonassa fossulata, Arenicolites* aff. *carbonarius, Arenicolites* isp., *Bergaueria perata, B.* cf. *radiata, Circulichnis ligusticus, C. montanus, Cochlichnus anguineus, C. luguanensis, Conichnus conicus, Cruziana problematica, Curvolithus multiplex, C. simplex, Curvolithus* isp., *Dendroidichnites* aff. *irregulare, Didymaulichnus miettensis, Dimorphichnus* isp. A, *Dimorphichnus* isp. B, cf. *Dimorphichnus* isp., ?*Diplocraterion* isp., *Gordia marina, Gyrolithes gyratus, G. scintillus, Halopoa imbricata, Helminthoidichnites tenuis, Helminthopsis abeli, H. hieroglyphica, H. tenuis, Monomorphichnus bilinearis, M. lineatus, M. needleiunm, Monomorphichnus* isp., *Palaeophycus annulatus, P. tubularis, Palaeophycus* isp., *Psammichnites gigas circularis, P.* cf. *saltensis, Rosselia erecta, Rosselia* isp., *Rusophycus avalonensis, Rusophycus dabardi, Rusophycus* isp., *Saerichnites kutscheri, Teichichnus rectus, Torrowangea rosei, Treptichnus bifurcus, T. coronatum, T. pedum, T. pollardi, Trichichnus linearis*, and *Trichichnus* isp. In comparison to the taxonomic revision done in Gougeon et al. [1], a few additional trace fossils are reported in the “Data 1” file in open nomenclature as their affinity is unclear (i.e. bilobed burrow, horizontal branching burrow, pyritized burrow, horizontal open spiral trail, undetermined burrow, and vertical burrow).

Body fossils are recovered from FH, GBH, LDC, and PM. These fossils correspond to (see also refs [1,9,13]): (1) two Ediacaran index fossils (*Harlaniella podolica* and *Palaeopascichnus delicatus*) reported from M1 and M2A at FH and GBH; (2) algal fossils (*Sabellidites cambriensis* and *Tyrasotaenia* sp.) found in M1, M2A, and M4 at FH, GBH, and PM; and (3) small shelly fossils (e.g. *Aldanella attleborensis, Watsonella crosbyi*) recovered from M3 and M4 at LDC (these are also present at FN as noted by Landing et al. [13]).

### 4.3 Methods: logging of strata and trace-fossil data points

Sections for each locality were selected either based on previous work (e.g. Myrow [8], Narbonne et al. [9]) or on geological maps of the area. Logging was done using a Jacob staff and reported on “Rite in the Rain” field notebooks.

For centimeter-scale logs (“Data 1” file), a stratigraphic interval to report data was delineated every 1 to 5 m. For each of these segments: (1) the interval was searched broadly for spotting the main sedimentary features and trace fossils; (2) a sedimentologic sketch of the interval was drawn on notebook (5 m of strata per page); (3) additional notes on sedimentologic features were reported (e.g. presence of syneresis cracks, tool marks); (4) bioturbation indices were evaluated (see below); (5) trace-fossil width and depth were recorded; and (6) additional notes on trace fossils were taken (e.g. recording of trace fossils on loose samples, or any unusual feature). For sedimentologic observations, grain-size scale for sandstone was based on Wentworth [14] and for mudstone on Lazar et al. [15]. Sedimentary structures are drawn as they are observed in the field, given a specific name (e.g. hummocky cross-stratification) where the characteristic features of the sedimentary structure are clearly visible. Ratios on sandstone/mudstone are kept accurate on the drawing but simplified in some instances, as the depositional rate of sand and mud laminae in M2 was notably too high to be reported exactly without a significant loss of time. Exact bed thickness of those deposits is, however, provided in millimeter-scale logs of the “Data 2” file.

Bioturbation intensity in cross-section view is reported using the Bioturbation Index (BI) of Taylor & Goldring [6], which consists of seven semi-quantitative categories: BI = 0 for no bioturbation (0 %); BI = 1 for sparse bioturbation (1–5 %); BI = 2 for low bioturbation (6–30 %); BI = 3 for moderate bioturbation (31–60 %); BI = 4 for high bioturbation (61–90 %); BI = 5 for intense bioturbation (91–99 %); and BI = 6 for complete bioturbation (100 %). Polished samples were used to check field observations and were assigned BI values. Bed surface bioturbation intensity is reported using the Bedding Plane Bioturbation Index (BPBI) of Miller & Smail [7] and consists of five semi-quantitative categories: BPBI = 1 for no bioturbation (0 %); BPBI = 2 for low bioturbation (1–10 %); BPBI = 3 for low to moderate bioturbation (11–40 %); BPBI = 4 for moderate to high bioturbation (41–60 %); and BPBI = 5 for intense bioturbation (61–100 %). Trace-fossil width and depth were reported using a manual caliper with a scale of 1 mm. The width was measured on burrows and trails exposed both on bed surfaces and vertical cross-sections. Where the width was uneven along the structure (e.g. for plug-shaped burrows *Bergaueria perata*), the measurement was taken at the widest portion. Burrow depth was reported from vertical exposures only and corresponds to the maximum extension of the visible trace fossil. No attempt was made at inferring a possible connection between a sandstone-filled vertical burrow with an overlying sandstone laminae or bed that does not show direct contact with the burrow (i.e. in the case of floating preservation-style of Droser et al. [10]). Trace fossils were first identified in the field and their features were secondarily double-checked using ca*.* 20,000 photographs. Ichnotaxonomy followed modern standards using ichnotaxobases and was done through an in-depth literature review (Gougeon et al. [1]).

For millimeter-scale logs (“Data 2” file), an interval 0.50–1.71 m thick was selected based on its remarkable exposure, accessibility, and its good representation of the sedimentologic characters of strata exposed. Emphasis was placed on strata from FH, considering its importance as a type section. Methods of investigation were slightly different than for centimeter-scale logs, and consisted of: (1) the delineation of an interval of analysis ca*.* 10 cm thick; (2) the exact and accurate report of sedimentologic features on logs drawn on a notebook (20 cm of strata per page): (3) the continuous report of BI along each log; (4) the report of the highest number of BPBI data points available; (5) the report of the stratigraphic position of trace fossils and their metrics; and (6) the report of additional information (e.g. presence of tool marks or body fossils).

### 4.4 Methods: sample preparation

Sixty-four samples were collected from FH, GBH, and LDC, encompassing the five members of the CIF. Each sample was coated using Epo Fix epoxy and was then heated. Samples were later cut with an electric saw and polished on a Hillquist 8-inch lapping machine using diamond-imbedded lapping discs. Digitalization of the sample surfaces was done using an Epson Perfection 4180 Photo scanner at high resolution (2500 ppp). The contrast of each digitalized surface was improved uniformly using “levels”, “contrast/brightness”, and “vibrance” tools on Adobe Photoshop (see also Gougeon et al. [2]). Ichnologic information coming from each of those samples was then reported on the “Data 1” file.

## Limitations

Gathering field datasets in sedimentology and ichnology is subjective and depends on the knowledge and time spent on each interval by the investigator. This bias can affect the quality of reports on bioturbation intensity data points (i.e. the evaluation of BI and BPBI), the “selection process” of each data point (i.e. if a data point will be reported at a specific stratigraphic level or not), and ichnotaxonomic decisions. This is notably relevant in stratigraphic intervals that display a high ichnologic content, in which some investigators will spend more time than others.

## Ethics Statement

This paper complies with the ethical guidelines of the publisher and did not involve experiments with human subjects and/or animals.

## CRediT Author Statement

**Romain Gougeon:** conceptualization, data curation, investigation, methodology, validation, visualization, writing – original draft, writing – review & editing, funding acquisition. **M. Gabriela Mángano:** conceptualization, methodology, writing – review & editing, supervision, funding acquisition. **Luis A. Buatois:** conceptualization, methodology, writing – review & editing, supervision, funding acquisition. **Guy M. Narbonne:** writing – review & editing, supervision, funding acquisition. **Brittany A. Laing:** investigation, writing – review & editing. **Maximiliano Paz:** investigation, writing – review & editing.

## Data Availability

Mendeley DataIchnologic and sedimentologic datasets from the Chapel Island Formation (Canada) (Original data)
